# Impact of genetic alterations on outcomes of patients with stage I nonsmall cell lung cancer: An analysis of the cancer genome atlas data

**DOI:** 10.1002/cam4.3403

**Published:** 2020-08-28

**Authors:** Song Xu, Yanye Wang, Fan Ren, Xiongfei Li, Dian Ren, Ming Dong, Gang Chen, Zuoqing Song, Jun Chen

**Affiliations:** ^1^ Department of Lung Cancer Surgery Tianjin Medical University General Hospital Tianjin China; ^2^ Tianjin Key Laboratory of Lung Cancer Metastasis and Tumor Microenvironment Lung Cancer Institute Tianjin Medical University General Hospital Tianjin China

**Keywords:** database, early stage, genomics, lung cancer, surgery

## Abstract

**Background:**

The prognostic factors for early‐stage nonsmall cell lung cancers (NSCLCs) are not well defined. This study aimed to investigate the effect of highly frequent mutations on the outcomes patients with early‐stage NSCLC, particularly those with surgically resected stage I disease.

**Methods:**

The Cancer Genome Atlas (TCGA) datasets for Lung Adenocarcinoma (LUAD), Lung Squamous Cell Carcinoma (LUSC), and Pan‐Lung Cancer (PLC) were accessed via cBioportal and searched to identify patients with stage I NSCLC. We identified candidate genes with a high (>10%) frequency of mutations and copy‐number alterations and examined their effect on overall survival (OS) and disease‐free survival (DFS). The details of clinicopathologic features were analyzed with the Fisher's exact， Mann‐Whitney *U* test and Cox regression analysis. Survival was analyzed with Kaplan‐Meier curves, and differences were compared with the log‐rank and chi‐square test.

**Results:**

We identified 408 patients with stage I NSCLC from the PLC dataset. Of the 41 candidate genes with high‐frequency mutation rates, six genes were significantly associated with OS: *TP53*, *LPP*, *MAP3K13*, *FGF12*, *BCL6*, and *TP63*. Further stratified analysis in PLC, LUAD, and LUSC datasets, we only identified that *TP53* was significantly associated with OS in patients with surgically resected stage I lung adenocarcinoma.

**Conclusions:**

*TP53* mutations are potentially markers of poor prognosis for stage I lung adenocarcinoma patients. The mutation status of this gene may contribute to clinical decision‐making with respect to selecting patients who may benefit from adjuvant therapy.

## Graphical abstract

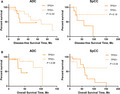

## 1 INTRODUCTION

Lung cancer is the leading cause of cancer mortality worldwide, representing about 13.2% of all new cancer cases and 25.9% of all cancer deaths.^1^ Although treatments have markedly improved, the prognosis for lung cancer remains very poor. Surgery is the first choice of curative treatments for medically operable patients with early‐stage nonsmall cell lung cancer (NSCLC). However, the 5‐year survival rate for these patients is about 71% to 83%.^2^ In addition, strong evidence supports adjuvant cisplatin‐based chemotherapy for patients with stage II and III NSCLC and squamous cell carcinoma (SqCC) or adenocarcinoma (ADC).^3^ For patients with stage I NSCLC, adjuvant chemotherapy is recommended only for the subset with high‐risk stage IB ADC, according to the National Comprehensive Cancer Network guidelines.^4^ For patients with stage I NSCLC, prognosis is favorable after surgical resection, but the recurrence rate ranges from 27% to 38%.^5^ Additionally, the time to disease recurrence varies markedly, even for patients matched by stage, histology, and treatment. These findings suggest high intertumoral heterogeneity of early‐stage NSCLC. Therefore, using only histologic classification and features is inadequate to determine treatment regimen and predict outcomes.

With significant advances in the understanding of the underlying mechanisms of lung cancer pathogenesis, attention has shifted from histologic to molecular classification of NSCLC. Genetic alterations not only guide the target therapy but also have prognostic implications. Some studies have demonstrated that *EGFR* and *KRAS* mutations were correlated with prognosis in early‐stage NSCLC,^6^, ^7^, ^8^ and numerous other oncogenic and tumor suppressor genes have been identified in the initiation and pathogenesis of NSCLC. Although many genes have been thoroughly investigated as biomarkers of treatment and prognosis in advanced NSCLC, their roles in the early‐stage NSCLC, particularly surgically resected stage I NSCLC, are not well understood.

The Cancer Genome Atlas (TCGA) is a project jointly supervised by the National Cancer Institute and the National Human Genome Research Institute in the United States, which aims to apply high‐throughput genome analysis technology for a better understanding of tumor biology and improvement of cancer treatment. Given the limited “real world” data available, we aimed to identify somatic mutations and corresponding clinical data from TCGA database. Not only have these analyses provided molecular markers for the prognosis of patients with early‐stage lung cancer, especially after surgical resection, but they may also help with clinical decision‐making with respect to identifying the subset of patients who could benefit from adjuvant therapy.

## 2 MATERIAL AND METHODS

The reporting of this study is in compliance with the STROBE (Strengthening the Reporting of Observational studies in Epidemiology) statement.^9^

### 2.1 Study design

**FIGURE 1:**
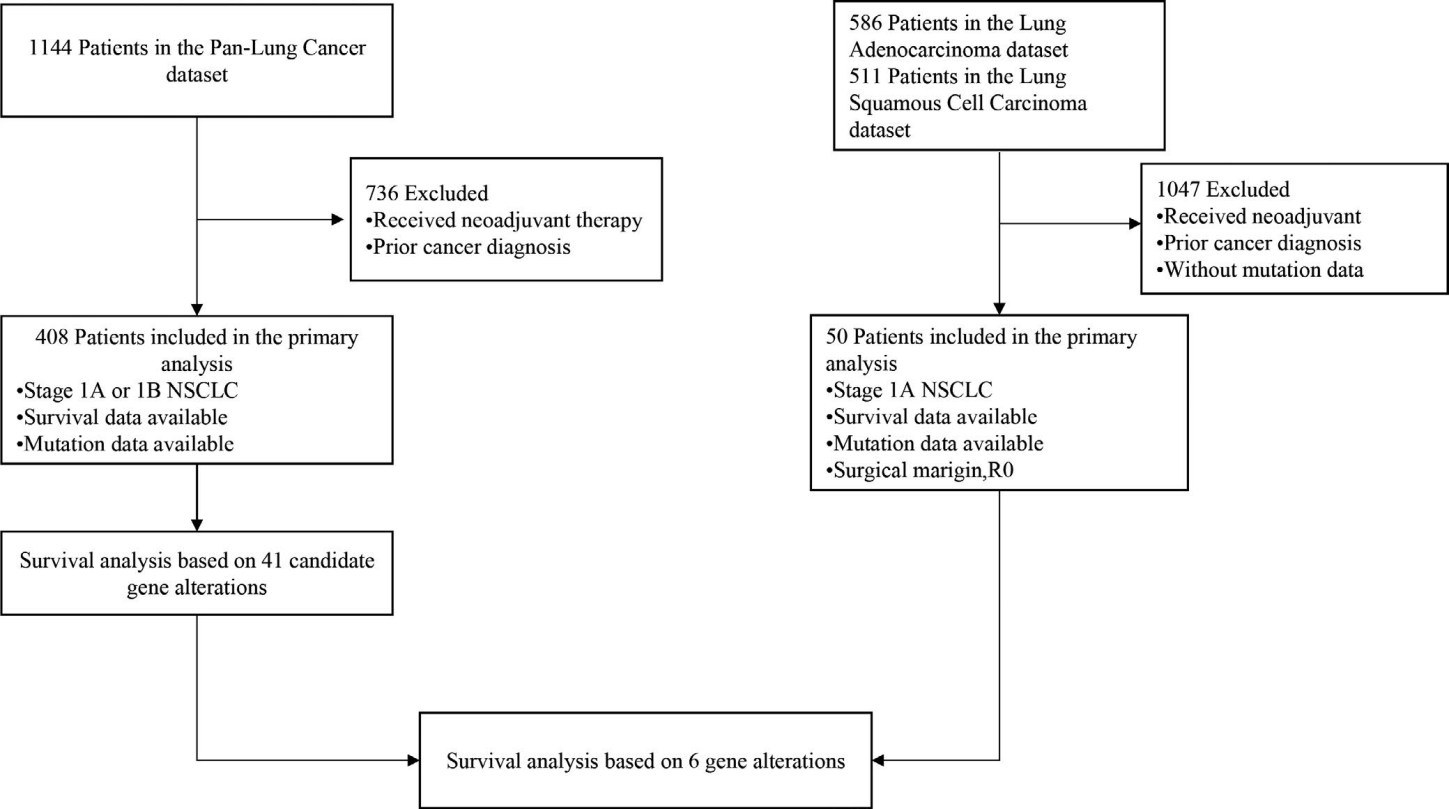
A flowchart of the retrospective study design

The cancer genomics and clinical profiles of patients with NSCLC were obtained from TCGA with an integrative analysis using cBioPortal bioinformatics tools (http://www.cbioportal.org/), as previously described.^10^, ^11^ Three cohorts were analyzed: Pan‐Lung Cancer (PLC, n = 1144), Lung Adenocarcinoma (LUAD, n = 586; Firehose Legacy), and Lung Squamous Cell Carcinoma (LUSC, n = 511; Firehose Legacy).^12^ PLC is a publically available dataset^13^ with the most comprehensive information about somatic genome alterations in lung ADC and SqCC. However, it lacks very detailed clinical information about surgical margin, adjuvant therapy, and DFS for patients with resected stage I NSCLC, whereas LUAD and LUSC provide this information. Clinicopathology, treatment details, and survival status were extracted, including sex, age, smoking status, tumor dimension, histology, total mutation count, surgical margin, prior cancer diagnosis, gene mutation and copy‐number alterations (CNA) type, disease‐free survival (DFS), and overall survival (OS). Clinical staging information was recorded with tumor‐node‐metastasis (TNM) classifications.^14^ The procedures of study design, including inclusion and exclusion criteria, are shown in Figure 1.

### 2.2 Statistical analysis

We compared baseline characteristics of patients with the Fisher's exact test for categorical data and the Mann‐Whitney *U* test for continuous variables, as appropriate. OS and DFS were estimated with Kaplan‐Meier curves, and differences were compared with the log‐rank and chi‐square test. The clinical Characteristics of *TP53*gene mutation were estimated by Cox regression analysis.*P* values less than .05 were considered statistically significant. Statistical analyses were performed with GraphPad Prism 7.0 (GraphPad Software, Inc).

## 3 RESULTS

### 3.1 Sequencing data and survival analysis of patients with stage I NSCLC

From the 408 patients with stage IA or IB NSCLC from the PLC dataset, we retrieved 41 candidate cancer genes with high (>10%) frequency rate (Table S1): 15 somatic mutations (Table S2) and 26 CNAs (Table S3). All candidate genes were analyzed to determine their association with OS.

**FIGURE 2:**
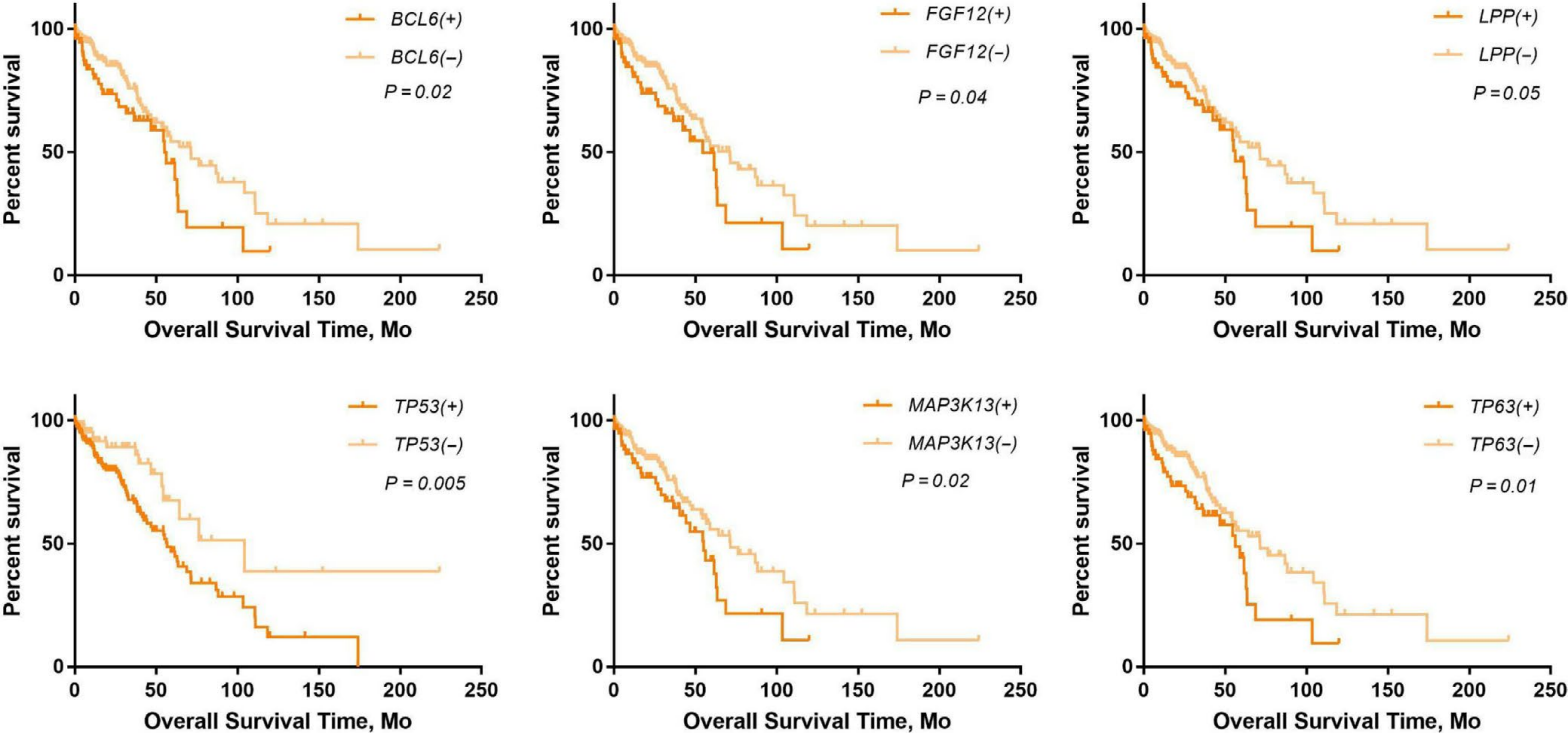
Survival analysis on genetic alterations. Six of 41 genes with high alteration rates (>10%) were significantly correlated with overall survival of 408 stage I NSCLC patients from the Pan‐Lung Cancer Dataset. The log‐rank test was used to compare survival curves

**FIGURE 3:**
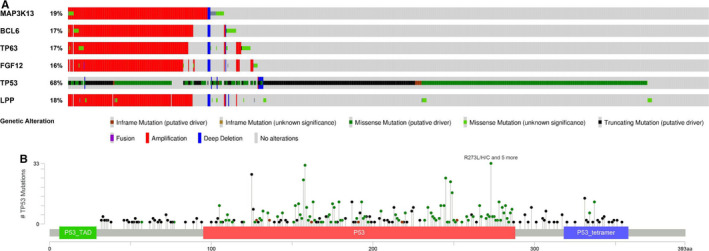
Characterization of genetic alterations and mutation map in stage I NSCLC. Data was from cBioPortal bioinformatics tools (http://www.cbioportal.org/ ). A, Six genetic alterations. B, Mutation map of *TP53*. The abbreviation “aa” indicates amino acid

Alterations in six genes (*TP53*, *LPP*, *MAP3K13*, *FGF12*, *BCL6*, and *TP63*) were significantly associated with the OS of patients with stage I NSCLC (Figure 2). Details of the six gene alterations are shown in Figure 3A *LPP*, *MAP3K13*, *FGF12*, *BCL6*, and *TP63* mainly had CNAs, *TP53* mainly had somatic mutations. Mutation maps of *TP53* are shown in Figure 3B. Moreover, we found that SqCC is the predominant histology in the *LPP*, *MAP3K13*, *FGF12*, *BCL6*, and *TP63*, but histology distribution was not significantly different for *TP53* (Figure S1).

**FIGURE 4:**
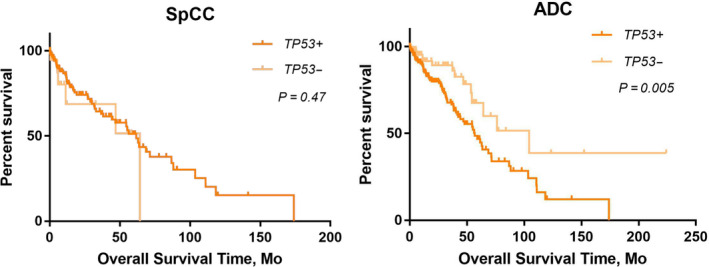
Overall survival rates of patients with stage I ADC or SqCC from the pan‐lung cancer dataset. The log‐rank test was used to compare survival curves. *TP53* mutant vs wild type. NSCLC, nonsmall cell lung cancer; SqCC, squamous cell carcinoma

In the PLC dataset, *TP53* was the most frequently mutated gene in stage I NSCLC, with five types of mutations at a total of 183 mutation sites (Table S4). We performed a stratified analysis of *TP53* on survival of lung SqCC or ADC. Interestingly, *TP53* mutations were associated with a worse OS in patients with stage I lung ADC, but they were not associated with OS in patients with lung SqCC (Figure 4). For *LPP*, *MAP3K13*, *FGF12*, *BCL6*, and *TP63* further stratified analyses did not show significant differences between patients with lung ADC or SqCC (data not shown).

### 3.2 TP53 mutations in patients with surgically resected stage IA NSCLC

In the 1093 patients from the LUAD and LUSC datasets, 50 patients with stage IA NSCLC who have mutation data available had undergone definite R0 surgical resection without prior cancer diagnosis occurence, and the clinicopathologic characteristics of the *TP53* mutations are shown in Table 1. The number of patients with mutated vs wild‐type *TP53* was comparable in this study cohort (n = 28 vs n = 22, respectively). However, the distribution of *TP53* mutation differed among patients with lung ADC vs SqCC, with the mutation being more common in lung SqCC. In addition, patients with the *TP53* mutation were more commonly heavy smokers and had a higher mutation burden (Table 1 ). And it was found that *TP53* mutation was an independent risk factor for the poor prognosis (Table S5).

**TABLE 1:** Clinicopathologic characteristics of patients with stage IA surgically resected NSCLC, stratified by *TP53* mutation status (n = 50)^a^

Characteristic	Mutated *TP53* (n = 28)	Wild‐type *TP53* (n = 22)	*P* Value
Female sex, No. (%)	18 (64.2)	15 (68.1)	>.99
Age, mean (SD), y	68.36 (6.15)	65.43 (8.6)	.726
Smoking, mean (SD), pack‐year	58.71 (18.94)	28.66 (18.81)	<.001
Smoking history (Never smoking)	1 (3.57)	3 (13.63)	.213
Tumor dimension, mean (SD), mm	11.5 (0.39)	10.4 (0.34)	.314
Histology, No. (%)			.04
Squamous cell carcinoma	17 (60.7)	4 (22.8)	
Adenocarcinoma	11 (39.3)	17 (77.2)	
Mutation count, mean (SD)	281.5 (189.3)	153.63 (99.0)	.004
Overall survival, median (range), months	42.2 (0.39‐151.15)	46.93 (2.6‐154.2)	.691

Abbreviation: NSCLC, nonsmall cell lung cancer.

^a^ Data from the lung adenocarcinoma and lung squamous cell carcinoma datasets.^13^

**FIGURE 5:**
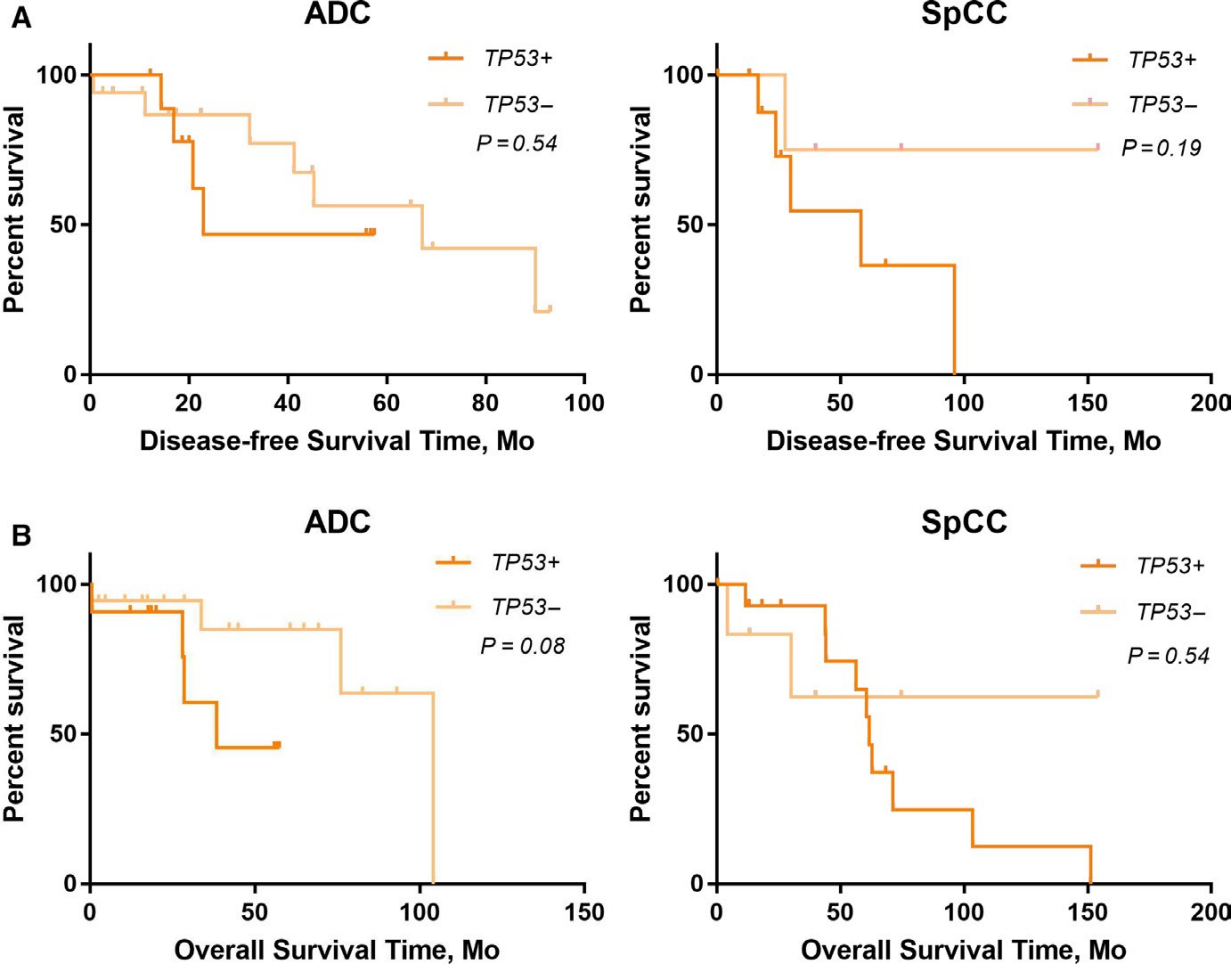
DFS and OS rates of patients with surgically resected stage IA SqCC or ADC, stratified by *TP53* mutation status. Patients with Stage IA SqCC or ADC were identified from the LUSC and LUAD datasets, respectively. A, DFS of patients with surgically resected Stage IA SqCC and ADC. B, OS of patients with surgically resected Stage IA SqCC and ADC. ADC indicates adenocarcinoma; DFS, disease‐free survival; NSCLC, nonsmall cell lung cancer; OS, overall survival; SqCC, squamous cell carcinoma

**FIGURE 6:**
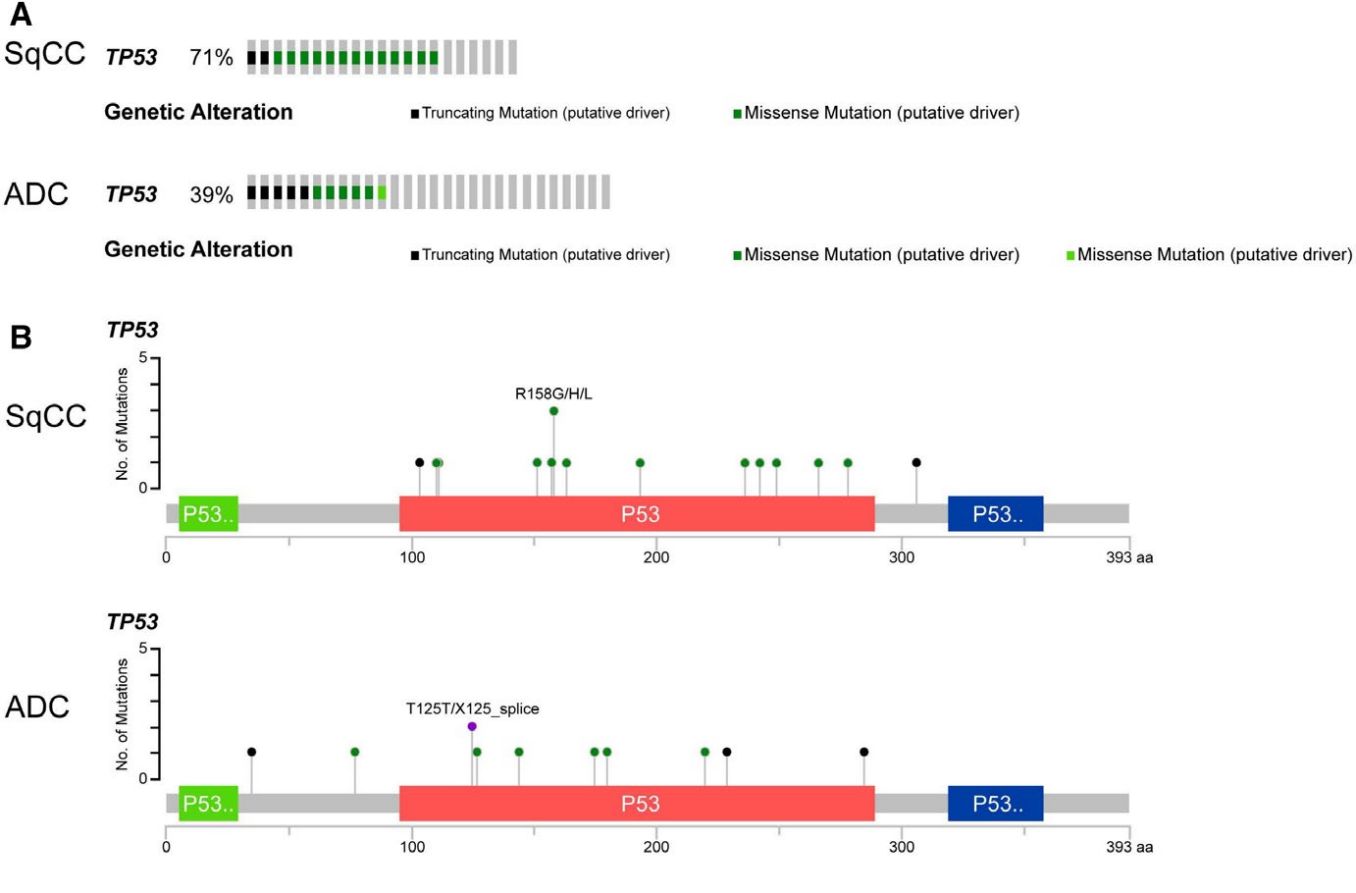
Genetic alterations (A) and mutation maps (B) of *TP53* in stage IA surgically resected ADC or SqCC. Data were from cBioPortal bioinformatics tools (http://www.cbioportal.org/). The abbreviation “aa” indicates amino acid; ADC, adenocarcinoma; SqCC, squamous cell carcinoma

To avoid the influence of postoperative adjuvant therapy on the survival, we only studied the role of *TP53* on the survival of 50 stage IA NSCLC patients. It was demonstrated that *TP53* mutations were not significantly associated with DFS in either lung ADC or lung SqCC (Figure 5A). However, consistent with the findings of total stage I NSCLC from the PLC dataset, *TP53* mutations were significantly associated with OS, but only for patients with lung ADC and not lung SqCC (Figure 5B). To further explore underlying mechanisms for the different roles of *TP53*, we compared the mutation types of *TP53* in lung ADC and SqCC. Lung SqCC had more *TP53* genetic alterations compared with lung ADC (Figure 6A), and the mutation sites (Figure 6B) of *TP53* were markedly different between lung ADC and SqCC (Table 2).

**TABLE 2:** *TP53* Mutations Identified in stage IA surgically resected NSCLC^a^, ^b^

Protein change	Type
Y236C	Missense
Y163C	Missense
P278S	Missense
H193R	Missense
C242F	Missense
R249W	Missense
R158H	Missense
V157F	Missense
L111R	Missense
R158L	Missense
G266V	Missense
R110L	Missense
R158G	Missense
P151R	Missense
I162dup	In_Frame_Ins
R306*	Nonsense
Y103*	Nonsense
R158Afs*12	Frame_Shift_Del
R175H	Missense
Y220C	Missense
Q144H	Missense
S127C	Missense
E180K	Missense
E285*	Nonsense
C229Yfs*10	Frame_Shift_Del
X125_splice	Splice_Site
L35Cfs*9	Frame_Shift_Del
T125	Splice_Region
P77L	Missense

Abbreviation: NSCLC, nonsmall cell lung cancer.

^a^ Data from the Lung Adenocarcinoma and Lung Squamous Cell Carcinoma datasets.^13^

^b^ Only one instance of each mutation was identified in the dataset.

Moreover, we also explored the roles of *LPP*, *MAP3K13*, *FGF12*, *BCL6*, and *TP63* in the survival of the 50 patients with stage IA resected disease in LUAD and LUSC datasets. However, none of these genes were significantly associated with survival (data not shown).

## 4 DISCUSSION

Currently, molecular examinations of gene mutations and CNAs are routinely performed in many centers for patients with advanced NSCLC but not those with resected stage I disease. From the PLC dataset, we selected 41 candidate genes with high (>10%) frequency rate in patients with stage I NSCLC, including 15 somatic mutations and 26 CNAs. Of these, we identified six genes that were associated with the OS of patients with stage I NSCLC. However, the PLC dataset lacks detailed clinical information about surgical margin, adjuvant therapy, and DFS for patients with resected stage I NSCLC. Therefore, to further explore the effects of the six genes on the prognosis after resecting stage IA NSCLC, we verified these findings by analyzing LUAD and LUSC datasets.

In the present study, we explored the role of *TP53* mutations on the prognosis of patients with stage I NSCLC by analyzing three public datasets from TCGA, in particular those patients with surgically resected stage IA disease. *TP53* might be a potential biomarker for the prognosis or the selection of adjuvant therapy for patients with early‐stage NSCLC. From the PLC dataset, we identified six gene alterations (*TP53*, *LPP*, *MAP3K13*, *FGF12*, *BCL6*, and *TP63*) that were significantly associated with OS. However, the PLC dataset lacked clinical information about surgical margin, adjuvant therapy, and DFS. Therefore, we used the LUAD and LUSC datasets to identify a cohort of patients with stage IA disease, R0 surgical margins, and no adjuvant therapy to examine the postoperative prognostic implication of genomic alterations. Only the *TP53* mutation was associated with worse OS, and this association was restricted to patients with lung ADC which was consistent with the findings in stage I NSCLC of PLC dataset. We noticed that there was a trend of lung ADC and SqCC patients with TP53 mutation having a worse DFS, however, without significance, which is probably due to the limited sample number in each group. In addition, we identified 183 *TP53* mutation sites, with five mutation types, in 408 patients with stage I NSCLC in the PLC dataset. Additionally, in the 50 patients from LUAD and LUSC datasets with stage IA resected NSCLC, the *TP53* mutation sites were different between patients with lung ADC vs SqCC. These differences might explain the discrepant impact of *TP53* mutation on lung ADC and SqCC.

*TP53* is one of the most frequently altered genes in NSCLC,^15^ and it has been associated with tumor risk, therapeutic outcomes, and survival prognosis in previous studies. Szymanowska et al^16^ showed that the *TP53* Arg72Pro polymorphism was associated with an increased risk of NSCLC. According to a pooled analysis of data from the International Adjuvant Lung Cancer Trial and three other randomized trials, the LACE‐Bio Collaborative Group reported that *TP53* mutations had no significant predictive value on the outcome of platinum‐based adjuvant chemotherapy. However, in patients who received adjuvant chemotherapy, *TP53* mutation was associated with shorter survival than wild‐type *TP53*.^17^, ^18^ A meta‐analysis of 19 studies reported that wild‐type *TP53* was associated with a significantly higher OS rate in all stages of NSCLC. They further demonstrated that the significant OS benefit in the *TP53* wild‐type group was limited to the subgroup with early‐stage (I or II) NSCLC and lung ADC.^19^ Consistent with the findings from our study, that meta‐analysis also indicated that *TP53* gene alterations suggested a poor prognosis in patients with early‐stage lung ADC. With respect to surgically resected early‐stage NSCLC, the previous reports are controversial. In 120 patients with NSCLC who underwent surgery, Mitsudomi et al^20^ found that a p53 mutation was a significant prognostic factor for worse survival in the patients with advanced disease (stages IIIA‐IV) but not early disease (stages I‐II). However, Ahrendt et al^21^ reported the opposite results; after analyzing 188 patients with operable NSCLC, they showed that the risk of death was significantly higher in patients with p53 mutations than wild‐type patients. Furthermore, a stratified analysis showed that the statistically significant prognostic effect (predicting worse survival) of p53 mutations was limited to patients with stage I NSCLC.^21^ Molina‐Vila et al^22^ reported that a *TP53* mutation was an independent predictor of shorter survival in patients with advanced NSCLC (stage IIIB‐IV), but they did not provide the data for patients with surgically resected early‐stage NSCLC.

What might explain these discrepant findings? We propose that these inconsistencies might be caused by the diversity of *TP53* genetic alterations. Lee et al^23^ did not find that patients with NSCLC and *TP53* mutations had a significantly worse survival compared to the wild‐type group, but a subgroup analysis showed that *TP53* variants with a high mutation frequency were associated with a significantly worse survival compared with wild type. From a multiregion sequencing study, Zhang et al^24^ identified a universal spatial heterogeneity of *TP53* mutation in NSCLC; therefore, the sites and timing of tumor acquisition may cause a marked difference in the positivity and frequency of *TP53* mutation. Moreover, Deben et al^25^ recently showed that the Pro allele of the *TP53* R72P polymorphism was predictive of worse OS in stage I NSCLC and that the R213R polymorphism was significantly associated with *TP53* mutations in patients with NSCLC ADC. Altogether, these data suggest that the diverse *TP53* genotypes may lead to the different p53 protein structures and complex intratumor genetic heterogeneity. These factors likely complicate the predictive power of *TP53* mutations on the prognosis of patients with NSCLC.

In addition, from the enrichment analysis from cBioPortal, co‐occurrence of *TP53* alterations was significantly associated with the amplification of *MAP3K13*, *FGF12*, *BCL6*, *LPP,* and *TP63* (Table S6). However, among these five genes, no other associations were identified, indicating that the copy‐number amplifications of *MAP3K13*, *FGF12*, *BCL6*, *LPP,* and *TP63* might be linked to or may occur downstream of *TP53* alterations. Tumor protein p63 (encoded by the *TP63* gene) is a member of the p53 family. Some previous studies showed that genetic variants in *TP63* were associated with an increased risk of lung adenocarcinoma,^26^, ^27^, ^28^ but genomic amplification of p63 is paradoxically associated with prolonged survival in NSCLC.^29^ The clinical significance and biologic roles of *LPP*, *MAP3K13*, *FGF12,* and *BCL6* in NSCLC, particularly early‐stage disease, remain unclear and require further investigation.

We acknowledge several limitations to this study. First, this was a retrospective study, and some clinicopathologic variables were not included in the TCGA database (there are no data on adjuvant therapy and surgical margin in the PLC dataset). Although the LUAD and LUSC datasets provided more surgical information, some confounding variables remained that were not considered in the analysis, including the nature of tumor (solid or ground‐glass opacity), histology subtypes (invasive or minimally invasive), growth pattern (lepidic or nonlepidic), and tumor grade (well or poorly differentiated). Second, although the LUAD and LUSC datasets included 1093 patients with NSCLC, the number of stage IA NSCLC patients with mutation information available was only 50, which may have not been a sufficiently large sample size to measure the impact of mutation genes on survival. Lastly, all the mutation information of TCGA database comes from Caucasian patients. Considering the genetic difference between races, the present findings might be not applied for Asian NSCLC patients.

In conclusion, our study shows that for patients with stage I ADC, *TP53* mutations confer an OS disadvantage compared with wild‐type *TP53*. For patients with stage I surgically resected ADC, a *TP53* mutation might be a potential molecular biomarker of prognosis that can be used with other histologic markers to guide decision‐making about adjuvant therapy. However, these findings warrant further investigation through large‐scale retrospective and prospective clinical studies.

## CONFLICT OF INTEREST

The authors declared that they have no conflict of interest to this work.

## AUTHOR CONTRIBUTIONS

Song Xu, Yanye Wang, and Fan Ren provided the conception and design of this study, and drafted the manuscript. Song Xu, Yanye Wang, Fan Ren, Xiongfei Li, Dian Ren, and Ming Dong collected and analyzed the data. Song Xu, Yanye Wang, Dong Ming, Gang Chen, Zuoqing Song, and Jun Chen contributed to data analysis and interpretations. Song Xu, Dong Ming, and Zuoqing Song gave financial support. Jun Chen and Gang Chen gave administrative support. All authors read and approved the final manuscript.

## Supporting information

Figure S1

Table S1

Table S2

Table S3

Table S4

Table S5

Table S6

## Data Availability

The data used to support the findings of this study are available from the corresponding author upon request.
