# Supplementary figures and images for: Impact of genetic alterations on outcomes of patients with stage I nonsmall cell lung cancer: An analysis of the cancer genome atlas data
Source: Cancer Med. 2020 Aug 28;9(20):7686–94. doi: 10.1002/cam4.3403 (PMC7571826; doi:10.1002/cam4.3403)

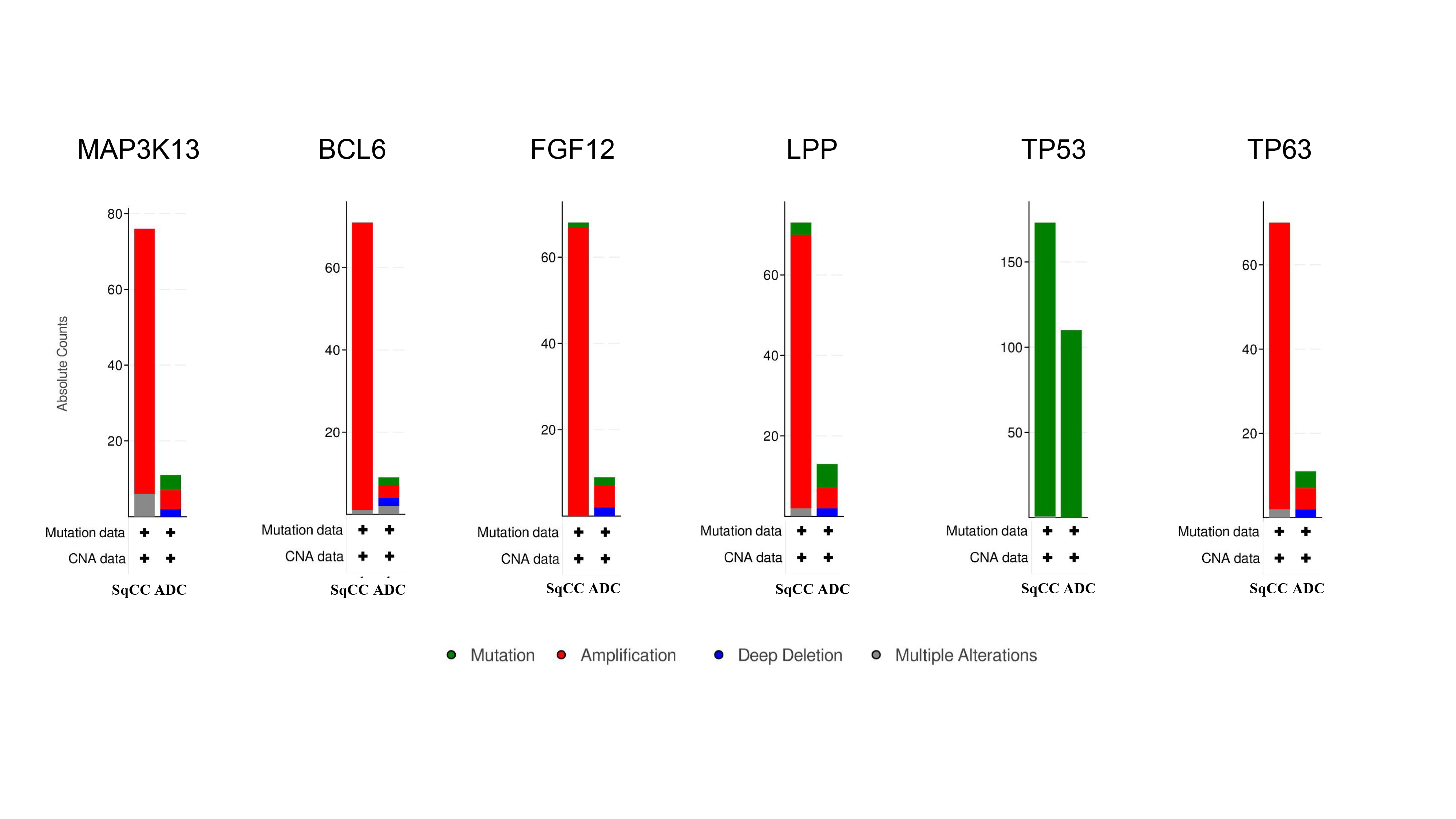

Supplement: Supplementary file 1 — Figure S1 [file CAM4-9-7686-s001.png]
