# Supplementary material for: Impact of genetic alterations on outcomes of patients with stage I nonsmall cell lung cancer: An analysis of the cancer genome atlas data
Source: Cancer Med. 2020 Aug 28;9(20):7686–94. doi: 10.1002/cam4.3403 (PMC7571826; doi:10.1002/cam4.3403)
Supplement: Supplementary file 2 — Table S1 [file CAM4-9-7686-s002.docx]

**Supplementary table 1. Candidate Genes With High (>10%) Mutation or Alteration Frequency Rate (n=41)**

| Gene | Somatic mutation | Copy-number alterations |
| --- | --- | --- |
| *BCL6* | √ |  |
| *CDKN2A* | √ |  |
| *CDKN2B* | √ |  |
| *CPS1* |  | √ |
| *DCUN1D1* | √ |  |
| *EIF4A2* | √ |  |
| *EPHA5* |  | √ |
| *ETV5* | √ |  |
| *FAT1* |  | √ |
| *FAT4* |  | √ |
| *FGF12* | √ |  |
| *GMPS* | √ |  |
| *KEAP1* |  | √ |
| *KLHL6* | √ |  |
| *KMT2C* |  | √ |
| *KMT2D* |  | √ |
| *KRAS* |  | √ |
| *LPP* | √ |  |
| *LRP1B* |  | √ |
| *MAP3K13* | √ |  |
| *MECOM* | √ |  |
| *MLF1* | √ |  |
| *MTAP* | √ |  |
| *NF1* |  | √ |
| *PCLO* |  | √ |
| *PDE4DIP* |  | √ |
| *PIK3CA* | √ |  |
| *PRKCI* | √ |  |
| *PTPRD* |  | √ |
| *RELN* |  | √ |
| *SDHA* | √ |  |
| *SOX2* | √ |  |
| *TBL1XR1* | √ |  |
| *TERC* | √ |  |
| *TERT* | √ |  |
| *TFRC* | √ |  |
| *TIPARP* | √ |  |
| *TP53* |  | √ |
| *TP63* | √ |  |
| *TRIP13* | √ |  |
| *WWTR1* | √ |  |
