# Supplementary material for: Impact of genetic alterations on outcomes of patients with stage I nonsmall cell lung cancer: An analysis of the cancer genome atlas data
Source: Cancer Med. 2020 Aug 28;9(20):7686–94. doi: 10.1002/cam4.3403 (PMC7571826; doi:10.1002/cam4.3403)
Supplement: Supplementary file 3 — Table S2 [file CAM4-9-7686-s003.docx]

**Supplementary table 2. Characteristics of Candidate Genes With High (>10%) Mutation Frequency Rate**

| **Gene** | **# Mutation** | **#Case** | **Frequency** |
| --- | --- | --- | --- |
| *TP53* | 290 | 283 | 69.40% |
| *LRP1B* | 218 | 147 | 36.00% |
| *PCLO* | 95 | 75 | 18.40% |
| *KMT2D* | 79 | 70 | 17.20% |
| *KRAS* | 71 | 69 | 16.90% |
| *RELN* | 86 | 66 | 16.20% |
| *KMT2C* | 67 | 59 | 14.50% |
| *FAT4* | 63 | 55 | 13.50% |
| *EPHA5* | 55 | 52 | 12.70% |
| *KEAP1* | 53 | 52 | 12.70% |
| *PTPRD* | 58 | 48 | 11.80% |
| *CPS1* | 50 | 46 | 11.30% |
| *FAT1* | 47 | 45 | 11.00% |
| *PDE4DIP* | 47 | 45 | 11.00% |
| *NF1* | 44 | 43 | 10.50% |
