# Supplementary material for: Impact of genetic alterations on outcomes of patients with stage I nonsmall cell lung cancer: An analysis of the cancer genome atlas data
Source: Cancer Med. 2020 Aug 28;9(20):7686–94. doi: 10.1002/cam4.3403 (PMC7571826; doi:10.1002/cam4.3403)
Supplement: Supplementary file 4 — Table S3 [file CAM4-9-7686-s004.docx]

**Supplementary table 3. Characteristics of Candidate Genes With High (>10%) CNA Frequency Rate**

| **Gene** | **Cytoband** | **CNA** | **#Case** | **Frequency** |
| --- | --- | --- | --- | --- |
| SOX2 | 3q26.33 | AMP | 92 | 22.50% |
| DCUN1D1 | 3q26.33 | AMP | 92 | 22.50% |
| KLHL6 | 3q27.1 | AMP | 88 | 21.60% |
| PIK3CA | 3q26.32 | AMP | 85 | 20.80% |
| MAP3K13 | 3q27.2 | AMP | 81 | 19.90% |
| PRKCI | 3q26.2 | AMP | 80 | 19.60% |
| TERC | 3q26.2 | AMP | 80 | 19.60% |
| TBL1XR1 | 3q26.32 | AMP | 80 | 19.60% |
| CDKN2A | 9p21.3 | HOMDEL | 79 | 19.40% |
| EIF4A2 | 3q27.3 | AMP | 78 | 19.10% |
| ETV5 | 3q27.2 | AMP | 78 | 19.10% |
| CDKN2B | 9p21.3 | HOMDEL | 77 | 18.90% |
| BCL6 | 3q27.3 | AMP | 76 | 18.60% |
| MECOM | 3q26.2 | AMP | 76 | 18.60% |
| LPP | 3q27.3-q28 | AMP | 75 | 18.40% |
| TP63 | 3q28 | AMP | 75 | 18.40% |
| TFRC | 3q29 | AMP | 74 | 18.10% |
| FGF12 | 3q28-q29 | AMP | 72 | 17.60% |
| TERT | 5p15.33 | AMP | 65 | 15.90% |
| SDHA | 5p15.33 | AMP | 64 | 15.70% |
| TRIP13 | 5p15.33 | AMP | 64 | 15.70% |
| MLF1 | 3q25.32 | AMP | 55 | 13.50% |
| TIPARP | 3q25.31 | AMP | 50 | 12.30% |
| GMPS | 3q25.31 | AMP | 49 | 12.00% |
| MTAP | 9p21.3 | HOMDEL | 48 | 11.80% |
| WWTR1 | 3q25.1 | AMP | 45 | 11.00% |

Abbreviations: AMP, amplification; CNA, copy number alteration; DEL, deletion.
