# Supplementary material for: Impact of genetic alterations on outcomes of patients with stage I nonsmall cell lung cancer: An analysis of the cancer genome atlas data
Source: Cancer Med. 2020 Aug 28;9(20):7686–94. doi: 10.1002/cam4.3403 (PMC7571826; doi:10.1002/cam4.3403)
Supplement: Supplementary file 5 — Table S4 [file CAM4-9-7686-s005.docx]

**Supplementary table 4. Characteristics of TP53 Mutation in Patients With Stage I Non−Small Cell Lung Cancer (Pan-Lung Cancer Dataset, n=183)**

| TP53 mutation | Type | #Mut | Poly-phen 2 |
| --- | --- | --- | --- |
| R158L | Missense | 6 | 0.988 |
| V157F | Missense | 6 | 0.998 |
| C242F | Missense | 5 | 1 |
| R273L | Missense | 5 | 0.994 |
| R282W | Missense | 5 | 1 |
| T125= | Splice_Region | 5 | NA |
| V73Wfs*50 | Frame_Shift_Del | 5 | NA |
| X307_splice | Splice_Site | 5 | NA |
| G154Afs*16 | Frame_Shift_Del | 4 | NA |
| R249S | Missense | 4 | 0.989 |
| X126_splice | Splice_Site | 4 | NA |
| Y163C | Missense | 4 | 0.999 |
| E298* | Nonsense | 3 | NA |
| G245C | Missense | 3 | 1 |
| G266V | Missense | 3 | 0.999 |
| G334V | Missense | 3 | 0.992 |
| H214R | Missense | 3 | 0.936 |
| L35Cfs*9 | Frame_Shift_Del | 3 | NA |
| P278A | Missense | 3 | 0.988 |
| P278S | Missense | 3 | 0.984 |
| Q192* | Nonsense | 3 | NA |
| Q317* | Nonsense | 3 | NA |
| R175H | Missense | 3 | 0.319 |
| R196* | Nonsense | 3 | NA |
| R248L | Missense | 3 | 0.995 |
| R337L | Missense | 3 | 0.946 |
| W91* | Nonsense | 3 | NA |
| X224_splice | Splice_Site | 3 | NA |
| X331_splice | Splice_Site | 3 | NA |
| Y220C | Missense | 3 | 0.998 |
| Y236C | Missense | 3 | 0.99 |
| C141W | Missense | 2 | 1 |
| C275F | Missense | 2 | 0.99 |
| E198* | Nonsense | 2 | NA |
| E271* | Nonsense | 2 | NA |
| E271K | Missense | 2 | 0.999 |
| E294* | Nonsense | 2 | NA |
| E68* | Nonsense | 2 | NA |
| G154V | Missense | 2 | 0.99 |
| G245V | Missense | 2 | 0.999 |
| H193L | Missense | 2 | 1 |
| P153Afs*28 | Frame_Shift_Ins | 2 | NA |
| R110L | Missense | 2 | 0.343 |
| R158Afs*12 | Frame_Shift_Del | 2 | NA |
| R213* | Nonsense | 2 | NA |
| R273P | Missense | 2 | 0.999 |
| R280I | Missense | 2 | 0.987 |
| R65Efs*58 | Frame_Shift_Del | 2 | NA |
| X125_splice | Splice_Site | 2 | NA |
| X187_splice | Splice_Site | 2 | NA |
| X225_splice | Splice_Site | 2 | NA |
| Y234C | Missense | 2 | 0.98 |
| A129Vfs*20 | Frame_Shift_Ins | 1 | NA |
| A159V | Missense | 1 | 0.772 |
| A276D | Missense | 1 | 0.986 |
| A69Gfs*80 | Frame_Shift_Ins | 1 | NA |
| A84Pfs*39 | Frame_Shift_Del | 1 | NA |
| C135F | Missense | 1 | 1 |
| C135Y | Missense | 1 | 1 |
| C176F | Missense | 1 | 0.998 |
| C229Yfs*10 | Frame_Shift_Del | 1 | NA |
| C238_M243del | In_Frame_Del | 1 | NA |
| C242G | Missense | 1 | 1 |
| D207Ffs*35 | Frame_Shift_Del | 1 | NA |
| D48Tfs*75 | Frame_Shift_Del | 1 | NA |
| D57N | Missense | 1 | 0.44 |
| E171* | Nonsense | 1 | NA |
| E180K | Missense | 1 | 0.989 |
| E224= | Splice_Region | 1 | NA |
| E224D | Missense | 1 | 0.23 |
| E285* | Nonsense | 1 | NA |
| E286* | Nonsense | 1 | NA |
| E286K | Missense | 1 | 0.999 |
| E286Q | Missense | 1 | 0.983 |
| E336* | Nonsense | 1 | NA |
| E339* | Nonsense | 1 | NA |
| E343* | Nonsense | 1 | NA |
| E346* | Nonsense | 1 | NA |
| E349* | Nonsense | 1 | NA |
| E62* | Nonsense | 1 |  |
| F109C | Missense | 1 | 0.999 |
| F212Sfs*3 | Frame_Shift_Del | 1 | NA |
| F270I | Missense | 1 | 0.996 |
| G105D | Missense | 1 | 0.996 |
| G105V | Missense | 1 | 0.998 |
| G244V | Missense | 1 | 1 |
| G245F | Missense | 1 | 1 |
| G245I | Missense | 1 | 1 |
| G266R | Missense | 1 | 0.951 |
| G279D | Missense | 1 | 1 |
| G279E | Missense | 1 | 1 |
| H115Vfs*28 | Frame_Shift_Del | 1 | NA |
| H168L | Missense | 1 | 0.661 |
| H179Q | Missense | 1 | 0.98 |
| H179Y | Missense | 1 | 0.991 |
| H193D | Missense | 1 | 1 |
| H193N | Missense | 1 | 1 |
| H193R | Missense | 1 | 1 |
| I162dup | In_Frame_Ins | 1 | NA |
| I195F | Missense | 1 | 0.984 |
| I195S | Missense | 1 | 0.995 |
| I232F | Missense | 1 | 0.937 |
| I232T | Missense | 1 | 0.67 |
| I251F | Missense | 1 | 0.998 |
| K132E | Missense | 1 | 0.994 |
| K164E | Missense | 1 | 0.964 |
| K291Sfs*51 | Frame_Shift_Del | 1 | NA |
| L111R | Missense | 1 | 0.997 |
| L130V | Missense | 1 | 0.999 |
| L194H | Missense | 1 | 1 |
| L252_T253delinsP | In_Frame_Del | 1 | NA |
| M237I | Missense | 1 | 0.999 |
| N131Qfs*17 | Frame_Shift_Del | 1 | NA |
| N239D | Missense | 1 | 0.991 |
| N239S | Missense | 1 | 0.772 |
| N247I | Missense | 1 | 0.998 |
| P128Lfs*42 | Frame_Shift_Del | 1 | NA |
| P151R | Missense | 1 | 0.996 |
| P152L | Missense | 1 | 0.886 |
| P152Q | Missense | 1 | 0.998 |
| P152Rfs*18 | Frame_Shift_Del | 1 | NA |
| P190L | Missense | 1 | 0.467 |
| P278H | Missense | 1 | 1 |
| P301Qfs*44 | Frame_Shift_Del | 1 | NA |
| P67Rfs*76 | Frame_Shift_Del | 1 | NA |
| P77L | Missense | 1 | 0.4 |
| Q136* | Nonsense | 1 | NA |
| Q136_C141del | In_Frame_Del | 1 | NA |
| Q144* | Nonsense | 1 | NA |
| Q144H | Missense | 1 | 0.99 |
| Q144P | Missense | 1 | 0.996 |
| Q144Tfs*5 | Frame_Shift_Ins | 1 | NA |
| Q165* | Nonsense | 1 | NA |
| Q192_H193del | In_Frame_Del | 1 | NA |
| Q331* | Nonsense | 1 | NA |
| Q354* | Nonsense | 1 | NA |
| R110_G112del | In_Frame_Del | 1 | NA |
| R156del | In_Frame_Del | 1 | NA |
| R158G | Missense | 1 | 0.994 |
| R158H | Missense | 1 | 0.45 |
| R175G | Missense | 1 | 0.99 |
| R209* | Nonsense | 1 | NA |
| R213L | Missense | 1 | 0.986 |
| R248Q | Missense | 1 | 0.994 |
| R248W | Missense | 1 | 1 |
| R249G | Missense | 1 | 0.994 |
| R249M | Missense | 1 | 0.868 |
| R249W | Missense | 1 | 0.838 |
| R267P | Missense | 1 | 0.999 |
| R273C | Missense | 1 | 0.999 |
| R273G | Missense | 1 | 0.997 |
| R273H | Missense | 1 | 0.643 |
| R273Lfs*72 | Frame_Shift_Del | 1 | NA |
| R273S | Missense | 1 | 0.997 |
| R282_R283del | In_Frame_Del | 1 | NA |
| R282H | Missense | 1 | 0.52 |
| R283P | Missense | 1 | 0.914 |
| R306* | Nonsense | 1 | NA |
| R333Vfs*12 | Frame_Shift_Del | 1 | NA |
| R337P | Missense | 1 | 0.99 |
| R65* | Nonsense | 1 | NA |
| S127C | Missense | 1 | 1 |
| S183* | Nonsense | 1 | NA |
| S315Lfs*30 | Frame_Shift_Del | 1 | NA |
| S46Tfs*5 | Frame_Shift_Del | 1 | NA |
| S94* | Nonsense | 1 | NA |
| T125P | Missense | 1 | 0.998 |
| T155I | Missense | 1 | 0.856 |
| T230Hfs*9 | Frame_Shift_Del | 1 | NA |
| V172D | Missense | 1 | 0.997 |
| V172G | Missense | 1 | 0.997 |
| V216E | Missense | 1 | 1 |
| V274D | Missense | 1 | 0.997 |
| W146* | Nonsense | 1 | NA |
| W53* | Nonsense | 1 | NA |
| X125_splice | Splice_Site | 1 | NA |
| X332_splice | Splice_Site | 1 | NA |
| Y103* | Nonsense | 1 | NA |
| Y126C | Missense | 1 | 1 |
| Y163D | Missense | 1 | 1 |
| Y205C | Missense | 1 | 0.998 |
| Y205F | Missense | 1 | 0.963 |
| Y234H | Missense | 1 | 0.971 |
| Y234S | Missense | 1 | 0.86 |

Abbreviation: del, deletion; FS, frame shift; IF, in-frame; ins, insertion.

(Poly-phen 2 score:Probably damaging interval value is 0.909-1; possibly damaging to 0.447-0.908; benign to 0-0.446)
