# Supplementary material for: Impact of genetic alterations on outcomes of patients with stage I nonsmall cell lung cancer: An analysis of the cancer genome atlas data
Source: Cancer Med. 2020 Aug 28;9(20):7686–94. doi: 10.1002/cam4.3403 (PMC7571826; doi:10.1002/cam4.3403)
Supplement: Supplementary file 6 — Table S5 [file CAM4-9-7686-s006.docx]

|  | Estimate | Hazard ratio (95%CI) | P Value |
| --- | --- | --- | --- |
| **Age（Mean）** | 68.55 |  | 0.757 |
| ＜70y | 23(46.0) | 0.859[0.325,2.250] |  |
| ≤70y | 27(54.0) | Ref |  |
| **Tissue Histology** |  |  | 0.433 |
| LUAD | 28(56.0) | 1.467[0.563,3.827] |  |
| LUSC | 22(44.0) | ref |  |
| **Longest Tumor dimension** |  |  | 0.452 |
| ≤1cm | 23(46.0) | 1.452[0.535,4.072] |  |
| ＞1cm | 27(54.0) | ref |  |
| **Mutation Count** |  |  | 0.137 |
| ≤182 | 24(48.0) | 2.048[0.796,5.272] |  |
| ＞182 | 26(52.0) | ref |  |
| **Smoking history** |  |  | 0.988 |
| Never | 4(8.0) | 0.851 | 0.876 |
| Current | 45(90.0) | 0.00 | 0.985 |
| NA | 1(2.0) | ref |  |
| **Gender** |  |  | 0.234 |
| Female | 33(66.0) | 0.521[0.178,1.528] |  |
| Male | 17(34.0) | ref |  |
| **TP53 mutation** |  |  | 0.015 |
| TP53(+) | 28(56.0) | 4.033(1.316,12.357) |  |
| TP53(wild) | 22(44.0) | ref |  |
| **Multivariate analysis** | TP53 | 4.033(1.316,12.357) | 0.007 |

**Supplementary table 5：****Cox regression analysis of TP53 gene mutation**
