# Supplementary material for: Impact of genetic alterations on outcomes of patients with stage I nonsmall cell lung cancer: An analysis of the cancer genome atlas data
Source: Cancer Med. 2020 Aug 28;9(20):7686–94. doi: 10.1002/cam4.3403 (PMC7571826; doi:10.1002/cam4.3403)
Supplement: Supplementary file 7 — Table S6 [file CAM4-9-7686-s007.docx]

**Supplementary table 6. Enrichment Analysis of *TP53* Mutations With *MAP3K13, FGF12, BCL6, TP63* and *LPP* (Data from cBioPortal, http://www.cbioportal.org)**

| **Gene** | **Cytoband** | **Percentage of alteration** | | **Log ratio** | **p-Value** | **q-Value** | **Direction/Tendency** |
| --- | --- | --- | --- | --- | --- | --- | --- |
|  |  | **Percentage of alteration**  **(Altered)** | **Percentage of Alteration**  **(Unaltered)** |  |  |  |  |
| *MAP3K13* | 3q27.2 | 79 (23.79%) | 8 (2.41%) | 2.502 | <0.001 | <0.001 | Co-occurrence |
| *FGF12* | 3q28-q29 | 69 (20.78%) | 8 (2.41%) | 2.237 | <0.001 | <0.001 | Co-occurrence |
| *BCL6* | 3q27.3 | 72 (21.69%) | 8 (2.41%) | 2.319 | <0.001 | <0.001 | Co-occurrence |
| *TP63* | 3q28 | 72 (21.69%) | 8 (2.41%) | 2.346 | <0.001 | <0.001 | Co-occurrence |
| *LPP* | 3q27.3-q28 | 76(22.89%) | 10(3.01%) | 2.078 | <0.001 | <0.001 | Co-occurrence |
